# Patient and healthcare professional perspectives on which potential prognostic factors for failure of total elbow replacement should be investigated

**DOI:** 10.1186/s13018-025-06186-0

**Published:** 2025-08-30

**Authors:** Zaid Hamoodi, Lianne Kearsley-Fleet, Jamie C. Sergeant, Adam C. Watts

**Affiliations:** 1 Upper Limb Unit, Wrightington Hospital, Wigan and Leigh Teaching Hospitals NHS Foundation Trust, Wrightington, Wigan, UK; 2 Centre for Epidemiology Versus Arthritis, Centre for Musculoskeletal Research, Division of Musculoskeletal and Dermatological Sciences, University of Manchester, Manchester Academic Health Science Centre, Manchester, UK; 3 Centre for Biostatistics, School of Health Sciences, Faculty of Biology, Medicine and Health, University of Manchester, Manchester Academic Health Science Centre, Manchester, UK; 4 Health Research Institute, Edge Hill University, Ormskirk, UK

**Keywords:** Elbow, Replacement, Patient and public involvement, Clinicians, Prognostic factors

## Abstract

**Background:**

Total elbow replacement (TER) is an established treatment for the painful arthritic elbow; however, TER has higher failure rates than other joint replacements, such as hip and knee replacement. Understanding the prognostic factors associated with failure of TER is essential for informed decision-making between patients and clinicians, patient selection, and service planning. The aim of this study is to explore the views of patients and healthcare professionals on which potential prognostic factors should be investigated in relation to TER failure.

**Methods:**

This evaluation comprised of two Patient and Public Involvement (PPI) workshops and a survey. PPI workshop 1 consisted of five PPI participants who helped to develop a survey assessing the importance of potential prognostic factors to investigate. The survey was shared electronically with members of the British Elbow and Shoulder Society (BESS) and clinicians internationally. In PPI workshop 2, 15 PPI participants listed factors they thought important to investigate, and 12 completed the survey.

**Results:**

Patients and healthcare professionals agreed that most factors in the survey should be investigated. Although this is not a comparative study, more of the healthcare professionals disagreed that ethnicity (49% v 33%) and VTE prophylaxis (42% v none) were important enough to be investigated, whilst more of the patients disagreed that socioeconomic status is important to be investigated (54% v 17%). Patients and healthcare professionals also suggested other factors not listed in the survey.

**Conclusions:**

Patients and healthcare professionals agreed on the importance of investigating most prognostic factors, but some factors were favoured by only one group. The results of this evaluation could help researchers decide which prognostic factors to investigate and which to routinely collect.

**Supplementary Information:**

The online version contains supplementary material available at 10.1186/s13018-025-06186-0.

## Background

A total elbow replacement (TER) is used to treat painful elbow conditions and restore function [1]. It is an important procedure in the management of end-stage elbow arthritis and severe trauma [2]. However, TER is not always successful and sometimes requires revision surgery to address complications [3]. Despite the importance of TER, little is known about which patients are more likely to require revision.

In order to investigate this further, potential prognostic factors associated with TER revision can be assessed. A prognostic factor is any variable associated with a risk of a particular health outcome amongst people with a given health condition [4]. For any complex intervention such as TER, prognostic factor research may explain the differences in outcomes between patients, which may then facilitate discussions about for whom and when TER should be used [5]. Prognostic research can also inform future research, for example, developing new interventions to target a modifiable prognostic factor or using a prognostic factor to stratify a population during study recruitment or analysis. Prognostic factor studies could also pave the way to developing a prognostic model in TER that could be used to make individualised risk predictions to guide clinical decision-making [5].

There are different types of prognostic factors in healthcare, such as patient characteristics, co-morbidities, symptoms, biomarkers, genetics, or treatment factors [4–6]. For outcomes of TER surgery, factors can be categorised as patient factors, factors related to the TER implant used, and factors related to how, where, and by whom the surgery was performed. It can be challenging to decide which potential prognostic factors to investigate and which factors to adjust for in statistical analyses, and most published studies investigating prognostic factors associated with TER failure lack information on why prognostic factors were investigated and how prognostic factors were selected to be adjusted for in the statistical analysis [7–18].

Like other research fields, prognostic factor research can benefit from the involvement of patients and healthcare professionals in formulating the research objectives and study methodology [19–22]. Patients are the recipients of the replacement surgery, and their input is necessary for understanding what factors they consider important. The experience of healthcare professionals is also vital as they are likely to have witnessed TER failure in the patient population and may be able to provide insight into which factors could potentially impact the need for revision surgery [23]. This information could guide researchers on which factors to investigate. The involvement of patients and healthcare professionals can also lead to research projects being accepted by those communities and help with bridging the research-practice gap in healthcare [24].

This study aims to elicit opinions from patients and healthcare professionals on which potential prognostic factors for TER failure (i.e. needing revision surgery) should be investigated, using a combination of a focus group and survey methodologies. The results from this study will be used to inform future prognostic factor research.

## Methods

**Fig. 1:**
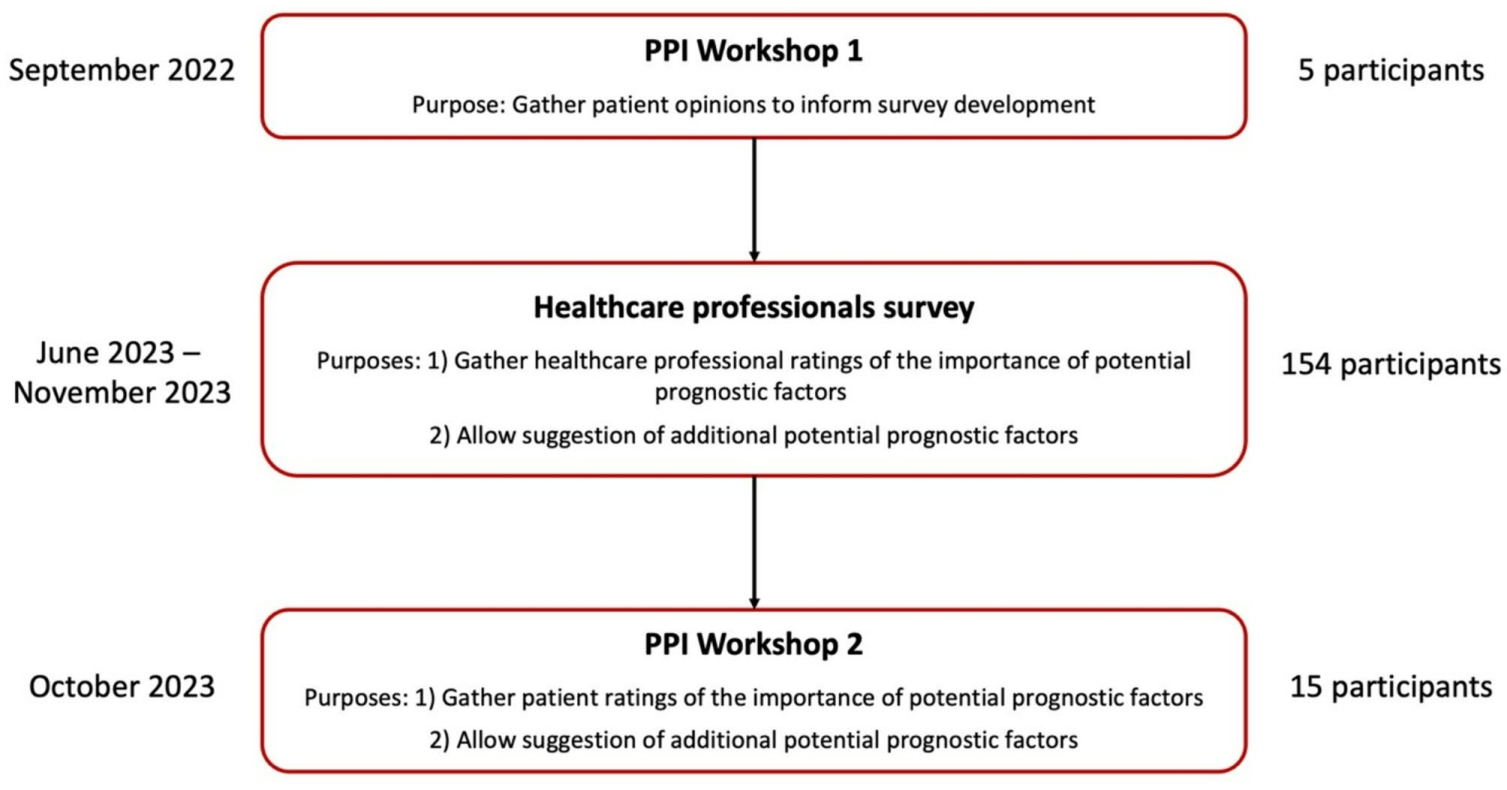
The structure of the study activities

This study used a combination of PPI focus group workshops and survey activities (Fig. 1). The PPI workshop activities included the PPI members at Wrightington, Wigan, and Leigh Teaching Hospitals NHS Foundation Trust (WWL). PPI members were contacted through the PPI team at WWL, and they include patients with lived experience of joint replacement surgery, including TER. The PPI team at WWL has been running for almost ten years and all members receive training in PPI activities when they join the group [25]. All the PPI members at WWL were contacted and had the opportunity to take part in both workshops. Both PPI group activities started with an introduction of all participants and researchers, followed by a presentation to introduce the study and the purpose of the meeting. The PPI activities are reported in line with the GRIPP2 (Guidance for Reporting Involvement of Patients and the Public) checklist [26]. All participants from the PPI were compensated for their time as guided by the National Institute for Health and Care Research (NIHR) payment guidance for researchers and professionals [27]. This was paid for by a grant awarded by the Dragons Den at the British Orthopaedics Trainee Association (BOTA) annual conference for PPI involvement.

This evaluation was part of a National Joint Registry (NJR) study that was approved by the NJR Research Committee [28]. The University of Manchester ethics decision tool, which determines whether a project requires formal ethical approval, was used and it confirmed that no formal ethical approval is required for this evaluation. All data were handled according to General Data Protection Regulation (GDPR) [29].

### PPI workshop 1

The first semi-structured PPI workshop activity was undertaken virtually in September 2022 using the Zoom (Version 5.16.2, San Jose, CA) platform and lasted 60 min. This PPI workshop centred on gathering patients’ opinions on which prognostic factors they considered important for investigation. Participants were informed that this information would be used to develop a survey to be shared with clinicians and with PPI participants in a second meeting.

### Survey activity

Two similar surveys were developed to evaluate the importance of different prognostic factors to (a) healthcare professionals and (b) patients as represented by PPI participants. These surveys were developed using the information gathered from PPI workshop 1, results from a systematic review investigating the prognostic factors associated with TER failure [30, 31]and insights from the authors’ clinical expertise (surveys are included in Supplementary File 1 and 2). Both surveys collected the same information regarding the importance of potential prognostic factors but collected different demographic data. Demographic data for healthcare professionals included their job titles and how many years they have been in clinical practice. Demographic data for PPI participants included age, sex, and if they had a previous joint replacement surgery. The anonymous surveys were administered using Qualtrics XM (Qualtrics, Provo, UT).

The information regarding prognostic factors was collected using a combination of Likert scale questions and free text boxes. The same prognostic factors were presented to healthcare professionals and PPI participants without making any assumptions regarding which factors are more likely to be relevant to clinicians or patients. The Likert scale questions included 24 factors, which were grouped into four categories: patient factors, implant factors, surgical factors, and surgeon/hospital factors. Healthcare professionals and PPI participants were asked to rate the importance of investigating the association between each potential prognostic factor and TER failure based on a 5-point Likert scale (Strongly disagree, disagree, neither agree nor disagree, agree, strongly agree).

Healthcare professionals had to choose one of the answers on the scale, whilst patients had an extra option “I don’t know what this is” if they did not know what the potential prognostic factor represented. The survey also had free text boxes, which could be used to add any further prognostic factors that healthcare professionals or PPI participants considered important to investigate.

The survey was shared electronically with healthcare professionals. A survey link was shared with members of BESS via email. This included surgeons and allied health practitioners involved in treating TER surgery patients. The BESS research committee shared the survey with their members on the 14th of June 2023. A reminder was sent on the 16th of June 2023. The survey link was also shared internationally on the 7th of July 2023, through an established WhatsApp group for elbow clinicians worldwide.

### PPI workshop 2

The second PPI workshop meeting was undertaken in October 2023 and lasted for 60 min. This meeting was held in person at WWL, although some participants joined online using the Zoom platform. Each PPI participant was asked to independently write a list of potential prognostic factors that they consider to be important, to allow them to consider factors important to them without being influenced by the contents of the survey. The participants were then asked to complete the survey for patients. Patients who attended the meeting face to face completed a printed version of the survey (*n* = 12). An electronic survey was shared with the PPI members who participated remotely, but these were not completed by them. By allowing patients to complete the survey during the PPI workshops, participants had the opportunity to ask what any of the prognostic factors were. In addition, they could choose the “I don’t know what this is” response option. For convenience, the “I don’t know what this is” response option will be labelled as “unknown” in this study.

### Data collection and analysis

PPI workshop meetings were facilitated by ZH, who made written anonymised field notes. The PPI lead at WWL chaired the meetings, and a research assistant produced a written record of the discussions.

Descriptive analyses were performed for the results from the PPI workshop and survey responses. The results from the Likert scale questions are presented as frequencies and proportions for each response option for each prognostic factor. The data collected from PPI workshop 2 and the free text in the survey were categorised into themes agreed upon by the research team. In the results section, the outcomes are presented in three categories: patient factors, implant factors and surgical factors. The surgical factor category includes two sections from the survey: surgical factors and surgeon/hospital factors.

PPI participants who chose the unknown option were excluded from the denominator used to calculate the proportion of patients who responded to that particular factor. The analysis was performed using R (Version 4.3.1).

## Results

There were five PPI participants who attended the first PPI workshop activity (using Zoom). All participants had a previous joint replacement surgery, although none had elbow replacement surgery. The second PPI workshop was attended by 15 PPI participants (12 face-to-face and three using the Zoom platform). None of the participants in PPI workshop 2 had participated in the PPI workshop 1 meeting.

The surveys were completed by 154 healthcare professionals (although six of these only partially completed the survey) and 12 PPI participants. The characteristics of those who participated in the survey are summarised in Tables 1 (healthcare professionals) and 2 (PPI participants).

**Table 1:** Characteristics of healthcare professional survey participants

**Characteristics**	Number of participants (*n* = 154)
**Job title/position:**	
Consultant surgeon	136 (88%)
Surgical trainee/resident	7 (6%)
Allied health practitioner	5 (3%)
Missing	6 (4%)
**Years in clinical practice**:	
1–5	20 (13%)
6–10	35 (23%)
11–20	47 (31%)
21–30	37 (24%)
> 30	9 (6%)
Missing	6 (4%)

**Table 2:** Characteristics of PPI survey participants

Characteristics	Number of participants (*n* = 12)
**Age**:	
51–60	2 (17%)
61–70	5 (42%)
> 70	5 (42%)
**Gender**:	
Female	11 (92%)
Male	1 (8%)
Nonbinary/third gender	0 (0%)
**Previous joint replacement**:	
Elbow replacement	5 (42%)
Other joint replacement	3 (25%)
None	4 (33%)

During the first PPI workshop, participants emphasised the importance of investigating patient and surgeon/hospital factors that could impact the failure of elbow replacement surgery. The patient factors that they highlighted included age, ethnicity, sex, co-morbidities, and the disease leading to the surgery (i.e. indication for surgery). The surgeon/hospital factors they highlighted included surgeon experience (i.e. how many years they have been practising surgery), the number of procedures the surgeon and/or hospital performs per year, and whether the surgery was performed in a hospital that specialises in joint replacements. All these potential prognostic factors were included in the survey. Some of the quotes from the patients in the initial patient involvement meeting were:

- “I would like to know if ethnicity would have an impact on the outcome of total elbow replacement surgery”.
- “I think the impact of age is very important to help decide if I would go ahead with the surgery at younger age or not”.
- “If the surgery was performed by an experienced surgeon, would this impact the outcome of the replacement surgery?”.

### Patient factors

**Fig. 2:**
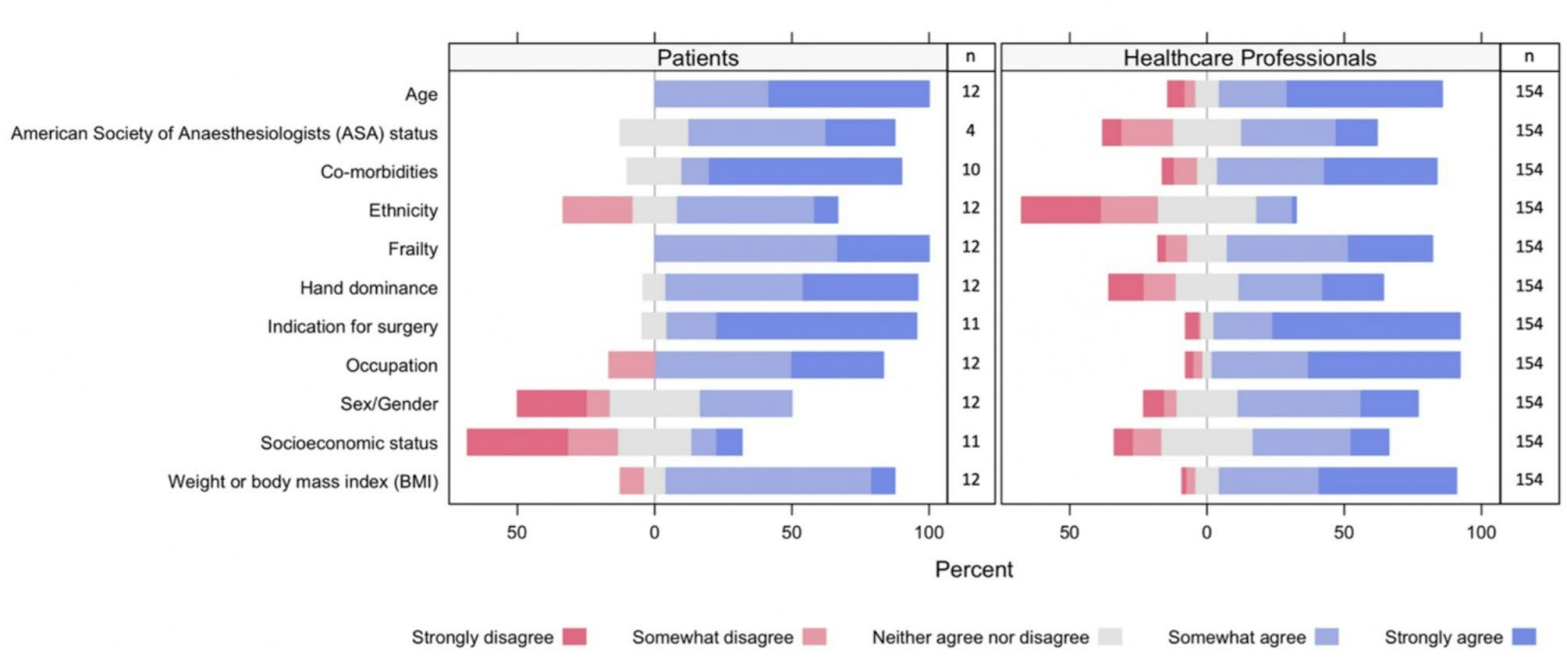
Patient and healthcare professional responses to whether the association between patient factors and failure of total elbow arthroplasty is important to be investigated (survey results)

All 154 healthcare professionals and 12 PPI participants submitted a response for each patient factor listed in the survey. Eight PPI participants chose the unknown option for ASA Physical Status Classification System, two chose it for co-morbidities, one for socioeconomic status, and one indication for surgery, and this is reflected in the denominator for these questions (Fig. 2). PPI participants agreed or strongly agreed that most factors other than socioeconomic status and sex should be investigated. Only two participants (18%) agreed (one agreed, one strongly agreed) that socioeconomic status should be investigated as a prognostic factor, and only four participants (33%) agreed that sex should be investigated.

In contrast, many of the healthcare professionals agreed or strongly agreed that socioeconomic status and sex should be investigated (49% and 66%, respectively). Ethnicity was the only patient factor for which more healthcare professionals disagreed or strongly disagreed (*n* = 76, 49%) than agreed or strongly agreed (*n* = 22, 14%) that it should be investigated as a prognostic factor. However, the majority of PPI participants (*n* = 8, 67%) agreed or strongly agreed that ethnicity is important be investigated as a prognostic factor.

In addition to the factors included in the survey, there were several other patient factors suggested in the free text by both healthcare professionals and PPI participants, including smoking status, alcohol consumption, medication use, psychological and mental health factors, use of walking aids, living status, whether the patient is a carer for any dependent person, and activity level before surgery. Other factors suggested by healthcare professionals alone included bone health status (most suggested factor, *n* = 11), followed by any previous elbow surgery (second most suggested factor, *n* = 10) and the type of surgery (third most suggested factor, *n* = 10). Healthcare professionals also suggested which specific co-morbidities should be examined, with diabetes being the most suggested disease (*n* = 4), followed by rheumatoid arthritis (*n* = 3) and previous trauma (*n* = 3) (Table 3).

**Table 3:** Other patient factors suggested by health care professional and PPI participants

Patient factors	Health care professionals *n* = 54	PPI participants *n* = 15
Co-morbidities:	11*	9*
• Diabetes (Hba1c level = 1)	4	1
• Medication use	3	5
• Previous trauma	3	3
• Rheumatoid arthritis	3	2
• Contralateral shoulder/elbow/wrist pathology	1	-
• Metabolic disease	1	-
• Neurological disorders	1	-
• Opioids use	1	-
• Renal	1	-
• Stickler syndrome	1	-
• Corticosteroid use	1	-
• Cardiac condition	-	1
• Depression and anxiety	-	1
Elbow specific factors	13*	13*
• Previous surgery and type	10	1
• Pre-operative elbow deformity and bone loss	1	-
• Previous elbow infection	1	-
• Skin condition	1	-
• Used of intra-articular steroid injection prior to surgery	1	-
• Range of movement before surgery	-	5
• The time had to wait to have surgery	-	1
• Preoperative pain level	-	13
• Strength pre surgery	-	1
Bone health	11*	3*
• Osteoporosis/Bone Mineral Density (BMD)	11	3
• Vitamin D level	1	-
Substance abuse	6*	4*
• Smoking	5	2
• Alcohol consumption	4	2
• Recreational drugs	1	1
Activity level	13*	10*
• Sporting activities (e.g., gold or tennis)	3	1
• Leisure activities	2	3
• Professional activity	2	-
• Activities of daily living	1	1
• Walking aides (e.g., crutches)	6	2
• Use of Wheelchair	1	-
• General physical health	-	5
• Gardening	-	1
• Employment status	-	1
• Time left until retirement	-	1
Psychological factors	7*	5*
• Can the patient understand what it means to live with total elbow replacement	2	3
• Pre-operative mental health/state	2	3
• IQ	1	-
• Understanding the limitations of own condition	1	1
• Psychological status	3	-
• Clinical team expectations	-	1
• Patient expectations	-	3
• Knowledge of how to care for new joint	-	4
Rehab and post operative advice	3*	5*
• Patient compliance	2	2
• Feedback following surgery	-	1
• Follow up quality (if follow up was prompt and how many times was the patient seen)	-	1
• Quality of Information regarding restriction about surgery	-	1
• Physiotherapist experience	-	1
• Language difficulties	1	-
Home circumstances	2*	6*
• Living alone	1	3
• Caring responsibility (caring after a child or dependant adult)	1	1
• Housing type (house, flat, bungalow, etc.)	-	3
• Support network for patient after surgery including at home (e.g., support at home to lift heavy items)	1	2
• Downstairs toilets	-	1

*represents the unique number of participants who suggested one or more item in this category

PPI participants also highlighted other patient factors which are important to patients, with pre-operative pain level being the most suggested factor (*n* = 13, 87%) (Table 3). Unlike the factors listed in the survey, which can be established prior to surgery, some of the factors suggested by PPI participants are typically not measurable at the time of surgery, such as the quality of the rehabilitation and the quality of the post-operative follow-up care. The patient’s compliance with post-operative advice is another factor that cannot be established prior to surgery and was suggested as an important factor by both healthcare professionals and PPI participants (Table 3).

### Implant factors

**Fig. 3:**
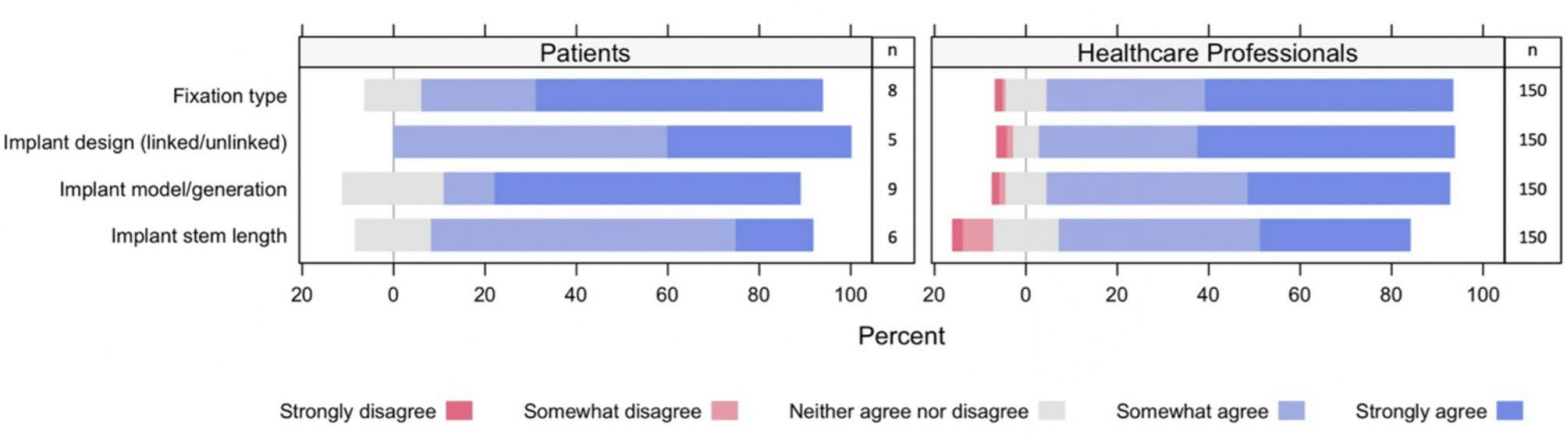
Patient and healthcare professional responses to whether the association between this implant factor and failure of total elbow arthroplasty is important to be investigated (survey results)

The section of the survey on implant factors was completed by 150 (97%) healthcare professionals and 12 (100%) PPI participants. Seven participants from the PPI group selected the unknown option for implant design, six for stem length, four for fixation type and three for implant model/design. Most healthcare professionals and PPI participants agreed or strongly agreed that all implant factors listed in the survey should be investigated. This included implant design, implant model, fixation type, and implant stem length (Fig. 3).

Healthcare professionals also suggested other implant factors that should be investigated such as the presence of an anterior flange on the humeral implant (*n* = 3) and the type of cement used (*n* = 2) (Table 4). The PPI participants suggested two other factors: the material (*n* = 1) and size (*n* = 1) of the implant used.

**Table 4:** Other implant factors suggested by health care professional and PPI participants

Implant factors	Health care professionals *n* = 10*	PPI participants *n* = 2*
Presence of anterior flange	3	-
Type of cement used (e.g., low viscosity cement)	2	-
Congruency of the implant articulation	1	-
Implant manufacturers	1	-
Length of the flange	1	-
Implant material/metal type	1	-
Number of implant size choices	1	-
Polyethylene design and thickness	1	-
Stem shape	1	-
Implant material/metal type	-	1
Size of the implant used	-	1

*represents the unique number of participants who suggested one or more item in this category

### Surgical factors

**Fig. 4:**
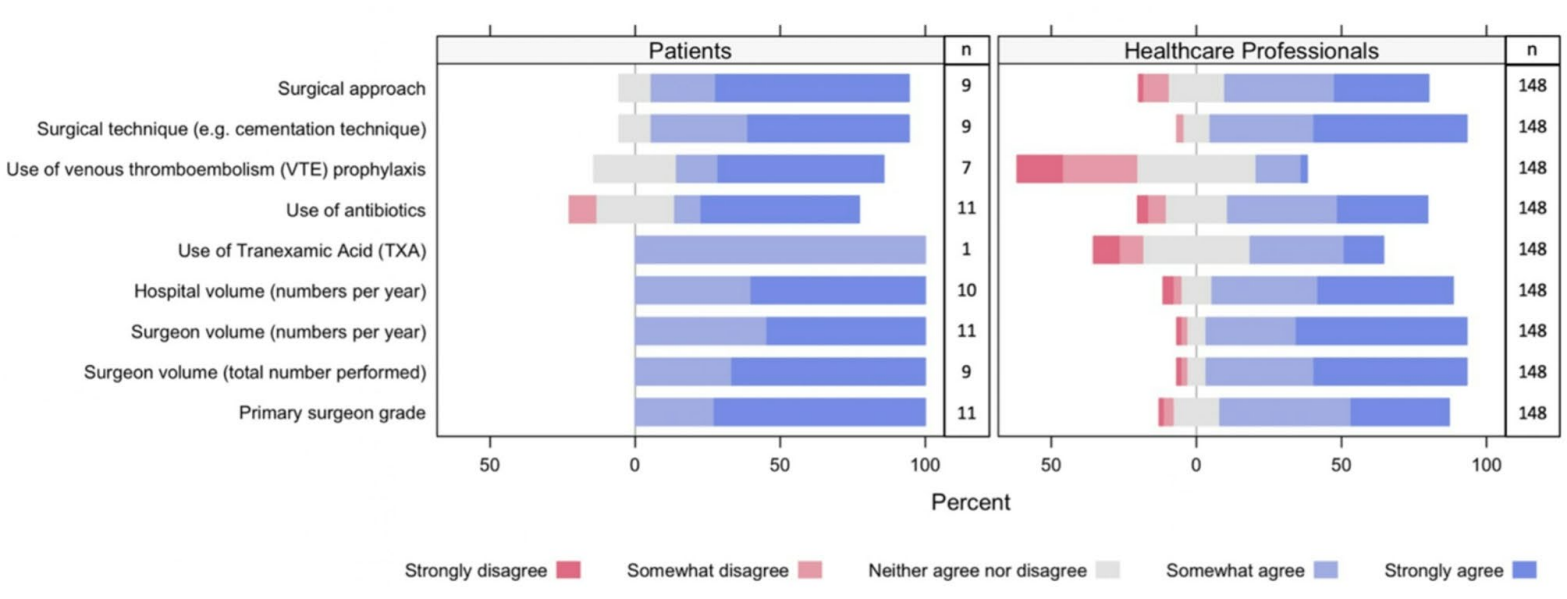
Patient and healthcare professional responses to whether the association between this surgical or surgeon/hospital factors and failure of total elbow arthroplasty is important to be investigated (survey results)

One hundred and forty-eight healthcare professionals (96%) responded to all the surgical factors listed in the survey and the majority agreed or strongly agreed that all bar one of those factors should be investigated. The only surgical factor that healthcare professionals predominantly strongly disagreed (26%), disagreed (16%), or neither agreed/disagreed (41%) should be investigated is the use of venous thromboembolism (VTE) prophylaxis (Fig. 4). Of the 12 PPI participants who responded to the surgical factors, 11 selected the unknown option for Tranexamic Acid (TXA), five for VTE prophylaxis, three for surgical approach, surgical technique, and surgeons’ volume, two for hospital volume, and one for the use of antibiotics, surgeon’s volume, and surgeon’s grade. Of the PPI participants who chose Likert response options, most agreed or strongly agreed that all the surgical factors listed in the survey were important to be investigated as prognostic factors.

Several other factors were suggested by health care professional and PPI participants, which were categorised into factors related to implant position, cementation of the implant, the use of antibiotics, intra-operative factors, surgeon/patient factors and other factors, as summarised in Table 5. Intra-operative factors and surgeon/hospital factors were the most suggested factors. Intra operative factors were suggested by 12 healthcare professionals whilst surgeon/hospital factors were suggested by six PPI members and six healthcare professionals. Implant positioning was suggested by eight healthcare professionals and cementation technique by six healthcare professionals.

**Table 5:** Other surgical factors suggested by health care professional and PPI participants

Surgical factors	Health care professionals *n* = 23	PPI participants *n* = 6
Intra-operative factors	12*	**0**
• Radial head replacement or excision or denervation	5	-
• Tourniquet use	3	-
• Collateral ligament repair	1	-
• Common flexor and common extensor origin repair	1	-
• Loan vs. own instruments	1	-
• Rotational stability	1	-
• The type of bone graft used (e.g., allografts vs. autograft)	1	-
• Ulnar nerve management	1	-
Implant positioning	8*	**0**
• Bone contact with anterior flange	3	-
• Implant alignment (Axis of components vs. axis of canal)	3	-
• Restoration of centre of rotation	3	-
• Radial column support/Lateralisation of the ulna humeral articulation	1	-
Cementation	6*	**0**
• Cementing technique	5	-
• If the cement was pressurised	1	-
• The use of cement plug	1	-
Antibiotics use	4*	**0**
• Antibiotics dose	1	-
• Antibiotics type	2	-
• Antibiotics duration	1	-
• The use of local antibiotics	1	-
Surgeon and hospital factors	6*	6*
• Surgeon’s training type (Academic, Public, or fellowship trained)	2	1
• Specialised vs. non-specialised orthopaedic hospitals	1	1
• If surgery performed at a major trauma centre	2	-
• Surgery out of hours	1	-
• Theatre team experience (e.g., specialised scrub nurse team)	2	-
• Hospital length of stay	-	1
• Type of anaesthetic used	-	2
• If the procedure was performed in the independent sector or NHS	-	1

*represents the unique number of participants who suggested one or more item in this category

## Discussion

Most published studies investigating prognostic factors associated with TER failure lack information on how the prognostic factors investigated were chosen. The choice of such prognostic factors may be based on the opinion of the research team and influenced by which variables are available in a dataset. As a result, the choice of those factors may be subject to the researchers’ personal biases and fail to account for the views of patients and healthcare professionals. The involvement of patients is likely to highlight factors that are important to them when discussing their treatment and rehabilitation with healthcare professionals and the input from healthcare professionals has the advantage of gaining insight from the clinical experience of the healthcare community. Therefore, knowledge of these factors could not only help inform research but also facilitate better-informed decision-making between patients and healthcare professionals.

This study shows that patients and healthcare professionals agree on the importance of investigating most of the factors that were included in the survey based on the published literature and PPI input. However, higher proportions of healthcare professionals than patients disagreed with the importance of ethnicity and higher proportion of patients disagreed that socioeconomic status should be investigated. Socioeconomic status and ethnicity may be used in medical research to check for inequalities by assessing disparity in access to services, such as replacement surgery [32–37]. Although most patients in this study disagreed that socioeconomic status is an important prognostic factor, they have suggested investigating factors which can be used to establish socioeconomic status, including occupation, living arrangements, and some educational elements [38]. Several studies have examined socioeconomic status as a prognostic factor in total knee replacement and total hip replacement and reported that lower socioeconomic status is associated with adverse events and worse patient-reported outcomes but there is no reported association with revision surgery [32–34, 39]. Evidence on the association between ethnicity and failure of TER has also been reviewed but the overall quality of evidence was ranked as very low. The understanding of patients’ and healthcare professionals’ perceptions of socioeconomic status and ethnicity, and the relationship between them, may benefit from further investigation [32].

Both healthcare professionals and patients suggested other factors not listed in the survey (i.e., not yet investigated and not highlighted by the first PPI workshop). Pre-operative pain level was suggested by most patients as an important prognostic factor to investigate. The association between pre-operative pain level and outcomes of TER is yet to be evaluated and could be addressed in future research. The most frequently suggested factors from healthcare professionals were bone health, previous surgery to the elbow, intra-operative factors, and the positioning of the implant. There is very low-quality evidence, from one study each, reporting previous surgery to the elbow and the positioning of the humeral implant to be associated with TER failure [14, 15]. The association between TER failure and the remaining factors is yet to be investigated. Studies are needed to evaluate the impact of factors that are deemed important by patients and healthcare professionals. The results from this study could be used to support researchers in choosing which prognostic factors to investigate and where to utilise available resources.

The results from this study may also support decisions around which data to routinely collect. Data on some of the prognostic factors in the survey are included in routinely collected data such as national joint registries: for example, the National Joint Registry (NJR) directly collects age, ASA, hand dominance, indication for surgery, sex/gender, weight or BMI, surgical approach, the use of VTE prophylaxis, primary surgeons’ grade, and implant fixation type. Other factors such as hospital/surgeon’s volume, implant design, and implant model can be indirectly collected or estimated. The results from this study suggest other prognostic factors that patients and healthcare professionals considered important, such as occupation, antibiotics use, tranexamic acid use, and implant stem length. However, adding more compulsory items to data collection might increase the burden on the organisation collecting the data, which may impact compliance. If the data collection is not compulsory, this might lead to a high proportion of missing data, as experienced with availability of data on weight and BMI in studies published from the NJR [40]. The other difficulty that can arise is how to classify the data collected. For example, what is the best method to classify the patient’s occupation so that it can be used meaningfully in research and care. Therefore, careful justification is required before it can be implemented on large scales such as joint registries. However, such factors could be collected and evaluated now in prospective cohort studies.

The study’s methodology was chosen pragmatically to gain insights from patients and healthcare professionals, though the approaches carried both limitations and advantages. There are limitations to the PPI workshop activities as they only included five participants in PPI workshop 1 and 15 participants in PPI workshop 2. Advertising the survey to a larger population of patients could have resulted in more participants, however, PPI workshops allowed more time for engagement and discussions with the participants, which meant that the researchers could better understand patient’s opinions. Also, several qualitative research studies suggest a group size of six to 15 participants is commonly used to ensure that all voices are heard, discussions remain manageable, and deep engagement is possible [41–43]. Additionally, logistical considerations (such as participant availability, time constraints, and the need for meaningful interaction) naturally limit the feasible size of such sessions. In contrast, using the survey to collect healthcare professionals’ input was an efficient method to involve many healthcare professionals from across the world and allowing for diverse perspectives; however, it was not possible to discuss the rationale for their selection of prognostic factors in depth. The free text boxes were added to capture additional information from healthcare professionals. Another limitation of this study is that it does not evaluate the perspectives of stakeholders and policymakers, who may perceive the value and impact of these prognostic factors differently. Future research should aim to address this gap.

Although PPI participants represented a variety of replacement surgeries, all but one participant were female, and they were all over the age of 50 years old. This study therefore does not necessary reflect the opinions of younger and/or male patients. There was a lack of participants with TER in PPI workshop 1, which may have resulted in the exclusion of important prognostic factors for individuals with TER during the survey development. To address this, participants in PPI workshop 2 were given the chance to discuss additional factors they considered important and to fill out free-text boxes in the survey, as detailed in this study. In the second workshop, only five members had undergone TER surgery. Nonetheless, the insights from patients without TER offer valuable validation for the study’s findings, as the perspectives of those with TER may be constrained by their individual experiences. In contrast, patients who have not undergone any TER surgeries can explore a broader range of prognostic factors that warrant investigation.

This study was able to capture the opinions from surgeons with different levels of experience as measured by the years in clinical practice, but other healthcare professionals only represented 5% of participants who completed the survey. Some results from healthcare professionals were also missing because 6 (4%) only partially completed the survey. Some of the results from the healthcare professionals were also missing because 6 (4%) only partially completed the survey. Additionally, to maintain the anonymity of PPI members and healthcare professionals, we refrained from collecting data on the ethnicity and socioeconomic status of PPI members, as well as the geographical demographics of healthcare professionals. Gathering this information would have enhanced our ability to assess the generalizability of the findings from this study.

## Conclusion

The involvement of patients and healthcare professionals experienced in joint replacement surgery is vital in understanding their views on which potential prognostic factors should be researched. The results of this study could support researchers and administrators in deciding which prognostic factors to collect data on, to investigate or to account for in analyses.

## Supplementary Information

Below is the link to the electronic supplementary material.

Supplementary Material 1

Supplementary Material 2

## Data Availability

No datasets were generated or analysed during the current study.

## Abbreviations

BESS: British Elbow and Shoulder Society
BOTA: British Orthopaedic Trainee Association
GDPR: General Data Protection Regulation
GRIPP2: Guidance for Reporting Involvement of Patients and the Public
NIHR: National Institute for Health and Care Research
NJR: National Joint Registry
PPI: Patient and Public Involvement
TER: Total elbow replacement
TXA: Tranexamic Acid
WWL: Wrightington, Wigan, and Leigh Teaching Hospitals NHS Foundation trust
VTE: Venous Thromboembolism
