# Supplementary material for: Patient and healthcare professional perspectives on which potential prognostic factors for failure of total elbow replacement should be investigated
Source: J Orthop Surg Res. 2025 Aug 30;20:808. doi: 10.1186/s13018-025-06186-0 (PMC12398077; doi:10.1186/s13018-025-06186-0)
Supplement: Supplementary file 1 — Supplementary Material 1 [file 13018_2025_6186_MOESM1_ESM.pdf]

# Default Question Block

## Study title: the predictors of failure in total elbow arthroplasty

Funders: The National Joint Registry (NJR), The Royal College of Surgeons of England (RCSEng), and The John Charnley Trust

Aim: the purpose of this study is to examine which prognostic factors might be associated with total elbow arthroplasty failure using the NJR data

Click here for abstract

Dear colleague,

We would be very grateful if you can complete this very short survey. The purpose of this survey is to establish which prognostic factors that might be associated with the failure and needing revision surgery of total elbow arthroplasty that surgeons and allied health professionals (AHPs) think are important and need investigating.

A prognostic factor in this study will be defined as any variable that is associated with a risk of failure leading to revision surgery in patients with total

elbow arthroplasty. The study will focus on prognostic factors that can be measured before or during surgery to support clinicians and patients in the decision-making and assessing the risk of failure associated with surgery. In this survey, several prognostic factors are grouped into patient factors, implant factors, surgery factors, and surgeon/hospital factors. If there are any other prognostic factors you think are important to investigate then please specify them in the free text boxes.

Many thanks

[redacted]

Participation: any surgeon or AHP who is involve in total elbow arthroplasty surgery/care can take part in this survey and the participation is voluntary. You are not required to provide any personal information such as name, address, phone number or email, and you don't have to answer any questions you don't want to.

Benefits and risks: there is no personal benefit from undertaking part in this survey. The data will be used to guide our research in which prognostic factors to include in our analysis. No risks are associated with this survey.

Confidentiality: no personal data are collected in this survey and the online survey system (Qualtrics) will not record email or IP (internet protocol) addresses. Therefore, responses will

remain completely anonymous. No one will be able to identify who has participated in this survey.

By choosing agree you consent to take part in this survey.

- ◯ Agree and proceed
- ◯ Disagree and stop

## The association between this **patient factor** and failure of total elbow arthroplasty is important to be investigated?

| | Strongly disagree | Somewhat disagree | Neither agree nor disagree | Somewhat agree | Strongly agree |
|---|---|---|---|---|---|
| Age | ◯ | ◯ | ◯ | ◯ | ◯ |
| American Society of Anaesthesiologists (ASA) status | ◯ | ◯ | ◯ | ◯ | ◯ |
| Co-morbidities | ◯ | ◯ | ◯ | ◯ | ◯ |
| Ethnicity | ◯ | ◯ | ◯ | ◯ | ◯ |
| Frailty | ◯ | ◯ | ◯ | ◯ | ◯ |
| Hand dominance | ◯ | ◯ | ◯ | ◯ | ◯ |
| Indication for surgery | ◯ | ◯ | ◯ | ◯ | ◯ |
| Occupation | ◯ | ◯ | ◯ | ◯ | ◯ |
| Sex/Gender | ◯ | ◯ | ◯ | ◯ | ◯ |
| Socioeconomic status | ◯ | ◯ | ◯ | ◯ | ◯ |
| Weight or body mass index (BMI) | ◯ | ◯ | ◯ | ◯ | ◯ |

Please list below any other patient factors you recommend investigating:

The association between this **implant factor** and failure of total elbow arthroplasty is important to be investigated?

| | Strongly disagree | Somewhat disagree | Neither agree nor disagree | Somewhat agree | Strongly agree |
|---|---|---|---|---|---|
| Fixation type | ○ | ○ | ○ | ○ | ○ |
| Implant design (linked/unlinked) | ○ | ○ | ○ | ○ | ○ |
| Implant model/generation | ○ | ○ | ○ | ○ | ○ |
| Implant stem length | ○ | ○ | ○ | ○ | ○ |

Please list below any other implant factors you recommend investigating:

The association between this **surgical factor** and failure of total elbow arthroplasty is important to be

investigated?

| | Strongly disagree | Somewhat disagree | Neither agree nor disagree | Somewhat agree | Strongly agree |
|---|---|---|---|---|---|
| Surgical approach | ○ | ○ | ○ | ○ | ○ |
| Surgical technique (e.g. cementation technique) | ○ | ○ | ○ | ○ | ○ |
| Use of venous thromboembolism (VTE) prophylaxis | ○ | ○ | ○ | ○ | ○ |
| Use of antibiotics | ○ | ○ | ○ | ○ | ○ |
| Use of Tranexamic Acid (TXA) | ○ | ○ | ○ | ○ | ○ |

Please list below any other surgical factors you recommend investigating :

The association between this **surgeon/hospital factor** and failure of total elbow arthroplasty is important to be investigated?

| | Strongly disagree | Somewhat disagree | Neither agree nor disagree | Somewhat agree | Strongly agree |
|---|---|---|---|---|---|
| Hospital volume (numbers per year) | ○ | ○ | ○ | ○ | ○ |
| Surgeon’s volume (numbers per year) | ○ | ○ | ○ | ○ | ○ |

| | | | | | |
|---|---|---|---|---|---|
| Surgeon’s volume (total number performed) | ○ | ○ | ○ | ○ | ○ |
| Primary surgeon’s grade | ○ | ○ | ○ | ○ | ○ |

Please list below any other surgeon/hospital factors you recommend investigating :

What is your position?

- ○ Surgeon/consultant
- ○ Surgical trainee/resident
- ○ Allied health professional (AHP)
- ○ Other

How many years have you been in clinical practice?

- ○ 1-5
- ○ 6-10
- ○ 11-20
- ○ 21-30
- ○ More than 30 years
